# Study of influential factors of provincial health expenditure -analysis of panel data after the 2009 healthcare reform in China

**DOI:** 10.1186/s12913-020-05474-1

**Published:** 2020-07-01

**Authors:** Jifei Hou, Liqi Tian, Yun Zhang, Yanzheng Liu, Jing Li, Yue Wang

**Affiliations:** 1 The Affiliated Hospital of Qingdao University, Qingdao, 266003 Shandong China; 2 Department of Medicine, Qingdao University, Qingdao, 266071 Shandong China; 3 Department of Research, Qilu Hospital, Cheeloo College of Medicine, Shandong University, Jinan, 250012 Shandong China

**Keywords:** Total health expenditure, Influential factors, Panel data, China

## Abstract

**Background:**

Total Healthcare Expenditure (THE) has increased substantially in all countries. Since the health system reform and health policy environment differ from each country, it is necessary to analyze the motivations of THE in a specific country.

**Methods:**

The objective of this study was to analyze the influential factors of Provincial THE (PTHE) per capita in China by using spatiotemporal panel data across 31 provinces (including provinces, autonomous regions, and municipalities, all called provinces in here) from 2009 to 2016 at the provincial and annual level. Generalized Estimating Equation (GEE) was used to identify the influential factors of PTHE per capita.

**Results:**

The number of beds per 10,000 population explained most of the variation of PTHE per capita. The results also showed that health expenditure in China reacts more to mortality compared with the Gross Domestic Product (GDP) per capita. But mortality and Out-Of-Pocket Payments (OOP) as a percentage of THE were associated with PTHE per capita negatively. The rate of infectious diseases and THE as a percentage of GDP had no statistical significance. And the Proportion of the Population Aged 65 and Over (POP65) impact PTHE per capita positively. But the coefficient was small.

**Conclusions:**

In response to these findings, we conclude that the impact of the increasing percentage of OOP in THE diminishes the PTHE. Furthermore, we find that both the “baseline” health level and health provision are positively correlated with PTHE, which outweighs the effect of GDP.

## Background

In China, Total Health Expenditure (THE) has grown considerably since the new round of health system reform started in 2009. Between 2009 and 2017, THE in China grew at a rate of 14.71% per annum, which is higher than that of GDP (11.30%), leading to an increase in THE share of GDP from 5.03 to 6.41% during the same period [1].

Since the mid-1990s, control of the rapid growth of THE has been one of the primary issues in health reforms. In 2009, the Chinese State Council announced a systematic plan to achieve universal health coverage by 2020 [2]. For a solid foundation of affordable, accessible, higher quality primary care, crucial questions remain to be the excess increase in THE.

The significance of understanding the main driving of THE is self-evident. For the government with limited resources could identify areas where future savings can be realized. The resulting estimates can also be useful for planning the future of the health care system in China. And the determinants of THE has become the subject of numerous empirical studies.

Among the influential factors of THE, the relationship between economic growth and THE per capita has been the most widely examined. It could be started by Newhouse [3], which concluded that 90% of variations of THE per capita at least can be explained by national income for developed countries. A recent study summarized 63 important empirical pieces of research which convinced a significant relationship between GDP and THE, and found that private health expenditures influence economic growth negatively while public health expenditures have a positive effect [4].

Later researchers analyzed both economic and non-economic factors (such as aging, disease, medical technology, health insurance, restrained public funding, increased health manpower, supply-side drivers, demographic structures, political determinants and characteristics of national health systems [5–8]). And to get a large study sample, most of those studies surveyed many countries and a long year simultaneously, usually concentrate on a pooled data of Organization for Economic Co-operation and Development (OECD) countries and started in twentieth century [9–14]. (And China is not a member of the OECD). Most of the results of previous studies concluded a positive association between these factors on THE. But a review of the literature on the determinants of healthcare expenditure concluded that no single pattern of results is clearly identified [15]. And there were little recent comprehensive evidence on whether and how economic and non-economic factors affect THE per capita by using spatial panel data in a specific country.

And a study by Herwartz [16] used pooled cross-section data and time series of OECD countries and recursive estimation procedures to show that there are divergences in the different health system, and emphasized the significance of country-specific effects for the study in health expenditure. Thus, the applicability of previous findings to the Chinese healthcare system is questionable and needs new specific empirical study. Furthermore, most earlier studies in China to identify the drivers of health expenditure growth were based on cross-sectional approaches for particular years, which starting time happened before a new health reform launched in 2009 [8]. And most of these studies aimed at national THE or provincial government health expenditure (PGHE), which are different from PTHE [17, 18]. PTHE consists of Out-Of-Pocket Payments (OOP), PGHE and social health expenditure (SHE).

The structure of our data called panel data allows us to test our hypothesis using dynamic econometric models and methods. Compared to traditional time-series data, panel data provide an advantage in determining the time-dependent effects in variables, simplifying computation and inference, and reducing the collinearity between variables [19–22]. Through econometric models and methods, which county-level fixed effects and year dummies are included to control for the county and year heterogeneity, the efficiency of estimates could be improved. And a spatiotemporal panel data model could gather more spatiotemporal dependence at the province and annual level [23].

So, this study aims to identify each selective driver’s contribution to PTHE growth since the new round of health system reform in 2009 by using spatial panel data across 31 provinces (including provinces, autonomous regions, and municipalities, all called provinces in here). And there are two main reasons to analyze factors from 2009. One hand, statistical data in 31 provinces are more comprehensive and available. On the other hand, the health system in China has undergone a huge change since 2009 [24], and previous statistics may not suitable for the current health situations and thus interferes with the results. For the detail information about this new reform could be found in other literature [2].

## Methods

### Data sources

The data of this study were extracted from the China Health Statistical Yearbook (2010–2018) and China Statistical Yearbook (2010–2018), which covered 31 provinces, autonomous regions, and municipalities. Those Yearbooks have collected extensive city-level information on health revenue and expenses, health service utilization, and medical facilities since 2003. Population aging was coming from the Chinese National Bureau of Statistics, whose statistical data covered various fields, from demographic, health, education, economic to industrial.

### Variables selection

Through the literature review and consider the availability of data, we have chosen the following variables. The definition of variables and their expected signs are in Table 1. For economic variables, we chose the GDP per capita and THE as a share of GDP to examine their relationship with THE. And we chose the out-of-pocket (OOP) payments as a percentage of THE as a financial protection factor. The demographic factor is the proportion of the population aged 65 and over (POP65), which is an indication of population aging. Epidemiological variables include mortality (MOR), which represents the “baseline” health level, and the incidence of infectious diseases (DIS) which is the incidence of Class A and Class B infectious diseases. We use the number of beds per 10,000 population (BEDS) as the health service supply, for the hospital in China always good at expanding, which performed in beds expansion. And it is the bed in all kinds of health care facilities, not only the number of beds in hospitals.

**Table 1:** Definition of variables

Factors	Variables	Expected sign
Economic factors	Gross domestic product per capita (GDP per capita)(USD)	+
	THE as a share of GDP (THE/GDP) (%)	+
Financial protection factor	Out-of-pocket payments as a percentage of THE (OOP/THE) (%)	–
Demographic factors	The proportion of the population aged 65 and over (POP65) (%)	+
Epidemiological factors	Mortality (MOR) (‰)	–
	The incidence of infectious diseases (DIS) (‰)	+
Health provision	The number of beds per 10,000 population (BEDS) (sheet)	+

### The model

In the literature, the dynamic panel model is frequently used to describe the longitudinal dependence of the response variable [25]. To examine the factors associated with the PTHE per capita, we consider in a first step: a basic model that takes the following form:

1 $$ {\displaystyle \begin{array}{c}{\mathrm{y}}_{\mathrm{i}\mathrm{t}}={\mathrm{y}}_{\mathrm{i},\mathrm{t}-1}\upalpha +{\mathrm{x}}_{\mathrm{i}\mathrm{t},1}{\upbeta}_1+{\mathrm{x}}_{\mathrm{i}\mathrm{t},2}{\upbeta}_2+\dots +{\mathrm{x}}_{\mathrm{i}\mathrm{t},\mathrm{p}}{\upbeta}_{\mathrm{p}}+{\upgamma}_{\mathrm{i}}+{\mathrm{e}}_{\mathrm{i}\mathrm{t}}\\ {}\mathrm{i}=1,\dots, \mathrm{N},\mathrm{t}=1,\dots, \mathrm{T}\end{array}} $$

where y_it_ is the response variable, y_i,t-1_ is the lagged value of the dependent variable. X_it_,j is the j th covariates, γ_i_ denotes unobserved time-invariant heterogeneity, and e_it_ is the noise term. In practice, this model could be efficiently tackled through GMM (Generalized Method of Moments) with an equivalent model:

2 $$ {\displaystyle \begin{array}{c}\Delta {\mathrm{y}}_{\mathrm{i}\mathrm{t}}={\mathrm{y}}_{\mathrm{i},\mathrm{t}-1}\alpha +\Delta {\mathrm{x}}_{\mathrm{i}\mathrm{t},1}{\upbeta}_1+\Delta {\mathrm{x}}_{\mathrm{i}\mathrm{t},2}{\upbeta}_2+\dots +\Delta {\mathrm{x}}_{\mathrm{i}\mathrm{t},\mathrm{p}}{\upbeta}_{\mathrm{p}}+{\mathrm{e}}_{\mathrm{i}\mathrm{t}}\\ {}\mathrm{i}=1,\dots, \mathrm{N},\mathrm{t}=1,\dots \kern1.75em ,\mathrm{T}\end{array}} $$

For (*Δ*) denotes the first difference. Where *Δ* y_it_ is the response variable, *Δ* y_i,t-1_ is the 1-order lagged term, *Δ* X_it_,_j_ is the j th difference of covariates [26].

Alternatively, some time-invariant country characteristics (geography, demographic) might correlate with the dependent variables, and the presence of the lagged explanatory variable y_it_ produced auto-correlation. Hence, to cope with this kind of problem, the following difference model is usually considered in practice,

3 $$ {\displaystyle \begin{array}{c}\Delta {\mathrm{y}}_{\mathrm{i}\mathrm{t}}=\Delta {\mathrm{y}}_{\mathrm{i},\mathrm{t}-1}\upalpha +\Delta {\mathrm{x}}_{\mathrm{i}\mathrm{t},1}{\upbeta}_1+\Delta {\mathrm{x}}_{\mathrm{i}\mathrm{t},2}{\upbeta}_2+\dots +\Delta {\mathrm{x}}_{\mathrm{i}\mathrm{t},\mathrm{p}}{\upbeta}_{\mathrm{p}}+{\upgamma}_{\mathrm{i}}+{\mathrm{e}}_{\mathrm{i}\mathrm{t}\kern1.75em }\\ {}\mathrm{i}=1,\dots, \mathrm{N},\mathrm{t}=1,\dots, \mathrm{T}\end{array}} $$

where γ_i_ denotes unobserved time-invariant heterogeneity, similarly [27]. This model manages to describe the longitudinal dependence of the response through the 1-order lagged terms {*Δy*_*i*, *t* − 1_}. However, it fails to directly specify the spatial dependence of response *Δ* y_i,t_ which could lead to confusing statistical interpretation.

In the paper, we employ the spatiotemporal panel model to analyze the complex data that simultaneously has longitudinal and spatial dependencies. Specifically, we consider the following spatiotemporal panel model:

4 $$ {\displaystyle \begin{array}{c}{\mathrm{y}}_{\mathrm{it}}={\upbeta}_0+{\mathrm{x}}_{1,\mathrm{it}}{\upbeta}_1+{\mathrm{x}}_{2,\mathrm{it}}{\upbeta}_2+\dots +{\mathrm{x}}_{\mathrm{p},\mathrm{it}}{\upbeta}_{\mathrm{p}}+{\mathrm{e}}_{\mathrm{it}}\\ {}\mathrm{i}=1,\dots, \mathrm{N},\mathrm{t}=1,\dots \kern1.75em ,\mathrm{T}\end{array}} $$

where y_it_ is the response variable, X_j,it_ is the j th covariates, and e_it_ is the noise term.

In particular, we assume:

e_t_ = (e_it_, …,e_Nt_), e_i_ = (e_i1_, …,e_iT_), and $$ \mathit{\operatorname{cov}}\left({e}_i\right)={\sigma}_0^2{R}_{cs}\left({\rho}_s\right) $$, $$ \mathit{\operatorname{cov}}\left({e}_t\right)={\sigma}_0^2{R}_{ar}\left({\rho}_t\right) $$.

where ${\sigma}_0^2$ is the variance of noise term, *R*_*cs*_(*ρ*_*s*_) is a (N*N) compound-symmetry structured correlation matrix with correlation coefficient *ρ*_*s*_, and *R*_*ar*_(*ρ*_*t*_) is a (T*T) order-1 autoregressive structured correlation matrix with correlation coefficient *ρ*_*t*_. That is, the noise term e_it_ is allowed to simultaneously have cross-sectionally and serially correlation. The correlation between provinces is assumed to be equal, while the correlation in time is assumed to be order-1 autoregressive.

In practice, we adopt the generalized estimating equation (GEE) method to estimate β [28],where the associated estimating equation is

$$ s\left(\beta \right)={x}^T{W}^{-1}\left(y- x\beta \right) $$

where y = (y_11_, …,y_1T_, …,y_N1_, …,y_NT_), *x* = (*x*_1_, …, *x*_p_), *x*_j_ = (*x*_11_,j, …, *x*_1T_,j, …, *x*_N1,j,_ …, *x*_NT,j_), and *W* = *R*_*cs*_(*ρ*_*s*_) ⊗ *R*_*ar*_(*ρ*_*t*_), and A ⊗ B is the Kronecker product of matrices A and B. The correlation coefficients *ρ*_*s*_ and *ρ*_*t*_ are artificially pre-determined before we solve estimating equation. *s*(*β*) = *o*, because a misspecified correlation structure of W does not influence the consistency of coefficient estimation [28, 29].

In this paper, we use PTHE per capita as dependent variables, for it offset the influence of demographics varied across provinces. PTHE per capita can be expressed as the product of these factors: POP65, MOR, GDP per capita, OOP/THE, THE/GDP, BEDS and DIS. Finial, we formalize the following dynamic regression model:

5 $$ {\displaystyle \begin{array}{l} lnPTHE={\beta}_0+{\beta}_1{lnGDP}_{it}+{\beta}_2 lnOOP/{THE}_{it}+{\beta}_3 lnTHE/{GDP}_{it}+\\ {}{\beta}_4\mathrm{lnPOP}{65}_{it}+\kern0.5em {\beta}_5 lnMORit+{\beta}_6\mathrm{lnD}{IS}_{it}+{\beta}_7\ln {BED}_{it}+{e}_{it}\end{array}}. $$

To offset the influence of inflation, all expenditure variables in the study were converted to 2009 yuan using the Consumer Price Index (CPI). Besides, all variables used in this analysis were log-transformed to deal with the heteroscedasticity between different variables. We assigned values to rhot and rhos with − 0.2 and 0.05 respectively, which maximizes the significance of the regression coefficient. Indeed, the consistency of the regression will not be affected if the choices of these two parameters are in appropriate intervals. The estimation procedures have been performed using the software R version 3.5.3. A two-sided *P* value of < 0.05 was considered statistically significant.

### Sensitivity analysis

To examine the stability of the regression results, a sensitivity analysis was undertaken to gain insight into the effect in the model of the level of uncertainty. We undertake sensitivity analysis by using variables before their log-transformed and employing a Generalized Method of Moments (GMM). We have reported the estimates and test results in appendix Table 1 and Table 2.

**Table 2:** Summary statistics based on 31provinces

Variables	Mean /Percentage	Std.	Min	Max
PTHE per capita ^a^	299.05	1, 236.77	107.03	1162.44
GDP per capita ^a^	5479.84	22, 182.79	1382.77	14,765.95
OOP/THE	32.49	7.74	5.46	45.80
THE/GDP	5.63	2.57	2.40	10.85
POP65	9.40	1.94	4.82	14.12
MOR	5.96	0.75	4.21	7.24
DIS	2.54	0.98	1.02	6.48
BEDS	44.57	10.37	23.85	75.48

^a^All monetary figures are in per capita USD at an average exchange rate of 2009–2016

## Results

### Descriptive statistics

Table 2 presents the results for our main study variables before their transformation into logarithms and is based on time series. We observed a wide variation on the depended variables in all the provinces, since the Std. Dev of PTHE per capita is over 100. On average, PTHE per capita accounts for $299.05. With a min value is $107.03 and a max value is $1162.44. A wider variation was also found on GDP per capita. And the mean value is $5479.84. OOP accounted for 32.49% of THE, while THE as a share of GDP is 5.63%. POP65 is 9.40%, and the max value is 14.12% in Chongqing province. The mean value of mortality and the rate of infectious diseases are 5.96‰ and 2.54‰, respectively. As for health supply, the number of beds per 10,000 population is 44.57 sheet.

### Regression results

The regression results presented in Table 3. For economic characteristics, we found a positive impact of GDP per capita on PTHE per capita, and the income elasticity was less than 1.00 (*p-value*≈0.00). OOP as a share of THE impact the PTHE per capita negatively ranged from − 0.40 to − 0.28 (*p-value*≈0.00). THE as a share of GDP has a positive impact but no statistically significance (*p-value* > 0.05). Regarding demographic factors, we observed a positive impact of population aging with a range from 0.02 to 0.28 (*p-value* < 0.01). Results also showed that there is a marginal negative correlation with no statistical significance between the rate of infectious diseases with PTHE per capita (*p-value* > 0.05). As for “baseline” health level and health supply, a strong correlation were found in the mortality, and the number of beds per 10,000 population, with PTHE per capita respectively (all *p-value* < 0.05). But the mortality impacts the PTHE per capita negatively.

**Table 3:** Panel estimation for GEE

Explanatory variables	Coef.	S. E.	*P* value	[95% CI]
In GDP per capita	0.45	0.06	0.00	[0.39 0.51]
In OOP/THE	−0.34	0.06	0.00	[−0.40–0.28]
In THE/GDP	0.06	0.10	0.12	[−0.04 0.15]
In POP65	0.15	0.12	0.01	[0.02 0.28]
In MOR	−0.58	0.19	0.00	[−0.77–0.37]
In DIS	−0.03	0.06	0.11	[−0.09 0.02]
In BEDS	0.59	0.10	0.00	[−0.49 0.69]

Number of observations = 248 Number of groups = 31

Dependent variable: ln PTHE per capita

## Discussion

### GDP per capita

GDP has been widely perceived as a determinant of the rapid growth for THE. A fair number of studies have analyzed the association between economic and THE. And almost all studies conclude a positive correlation [30–32]. But the income elasticity differs from each other, for there might be some missing variables in different studies and the methodology differ from each other. As the economy grows, access to health care improved and awareness of healthcare increased, resulting in a higher THE inevitably.

### OOP/THE

Our empirical results have shown that OOP as a share of THE is negative related to PTHE, and the elasticity of estimates is approximate to GDP. It means that with the increasing percentage of OOP in THE, less total health spending. It is a normal phenomenon that people are not willing to spend much when they have a large share of payment on themselves. However, if we raise the level of OOP, people faced with financial problems will gain access to necessary care hardly. And more OOP spending usually means better health outcomes [33]. The Chinese government needs to allocate the share of OOP on THE carefully and appropriately to ensure a reasonable increase in THE. It is contradictory and important for policymakers to decide the proper distribution of OOP on THE.

### THE/GDP

We have expected a positive impact of THE as a share of GDP on PTHE per capita. But there is no statistical significance with a *p-value* more than 0.05. We reviewed the original data, found that not all THE as a share of GDP increased year by year in 31 provinces. The variations in data are at uneven levels. It may be related to different economic development among provinces. And to a degree, it is the local government to decide the health investment. And even if the share of GHE on GDP increased, the THE account for GDP may not increase because of the increasing OOP or slowing growth of GDP. Further analysis needs to classified the provinces according to their economic level.

### POP65

Population aging is a universal phenomenon affecting all countries, though the internal mechanism differs from each other. Due to increasing life expectancy and high end-of-life cost which will lead to an increase in the average THE, it is usual to blame escalating THE on the population aging. Yet even here, we only found a modest positive relationship between POP65 and PTHE per capita, which is consistent with the findings of Gerdtham [34] and Dormont [35]. But a study from Italy concluded that the population aging impact THE positively which exceeds life expectancy and per capita GDP [36]. And a study from China also found a great regression coefficient and a fairly high significant level [37]. However, Kocot [6] concluded different types of THE react to aging diversely and the expenditure will be increasing for the 9 types of care and decreasing for 4 of them. Furthermore, studies have shown that the influence of aging on THE will decrease when controlled the time to death [38, 39], and a lower proportion of national health care expenditure will be spent on the high cost older compared with the middle age groups [40], implying that people are willing to devote more resources to those who benefit most, i.e. younger people than those high cost older people. The influence of aging on THE man be not as big as we thought, and the impact estimates various for different countries. A focus on population aging should be concerned with the elderly who are in poor attainable for health services. We need more studies to analyze the long and future influence of aging on THE in different countries.

### Mortality

We regard crude mortality rates as the output of health care, though not perfect. Our study exhibits a strong negative correlation between mortality and PTHE per capita. It shows that with the increase of health input, the result of health output is also improved. This is consistent with a panel data analysis from the SAARC-ASEAN region, which found THE had a significant effect in reducing infant mortality rate and private health care expenditure significantly decreased the crude death rate [41]. Other panel data analysis also concluded that private health spending has on average a higher health-promoting effect than public health spending [42]. But public health expenditure showed the opposite result, which shown that improper utilization of public sector funds and the presence of countries with ineffective governments may be the reason behind this.

However, Rothberg et al [43] conducted a study in the US and Lippi et al [44] in the European Union, all found that nearly no correlation between death ratio and health care expenditure. This may have important implications for the health department. It’s not like the more it spends, the better the healthcare outcome will be. Hence, the results of these studies have been inconsistent. It may be that the selection of representative indicators of health outcomes and statistical methods of mortality are various among different countries, and the estimation techniques used were also different. Furthermore, health input and output are complex, and only one or two indicators cannot be measured. We prefer to take MOR as a “baseline” health level. Thus, findings of the cross-country panel studies should be interpreted with caution. And further studies are needed to determine the reasons for this controversial relation between THE and health outcomes (including maternal, infant, and under-five mortality rates, crude death rates, life expectancy, and so on).

### The incidence of infectious diseases

The results showed that infectious diseases impact PTHE per capita negatively. But there is no statistical significance with the estimation, implying that little correlation between two variables. But a study from China that analyzed the policy-relevant drivers of THE from the perspective of disease and found a reduction in disease prevalence rates could reduce the growth rate of HE by 0.3 percentage points [8]. This may be because this research is based on the analysis of China’s cross-sectional data from 1993 to 2012. And in the early days, infectious diseases in our country were more serious. The incidence of infectious diseases in all provinces of China has been declining year by year since the new medical reform in 2009. And China has promulgated a series of regulations and laws to reduce the incidence of infectious disease and acquired some achievements. However, it should also be noted that the incidence of infectious diseases is more related to preventive health expenditure than to THE in China, and provinces with high THE do not necessarily invest more preventive health resources. This may account for a weak relationship between the incidence of infectious diseases and THE.

However, as the disease spectrum changes, the incidence of infectious diseases has decreased gradually and chronic non-communicable disease (CND) increases year by year. A new systematic analysis studied the top-10 leading causes of death and DALYs at the national level in China, found that CND and road traffic injuries were the main reasons [45]. For CND with a high cost but hard to cure, we should spend more on early prevention rather than the stage of illness. As for traffic injury, relevant regulations, such as fast seatbelt, no drinking when driving, baby safety seat, may be more useful than simple health care spending. New emerging challenges about CND need to be settled in China. But the recent global outbreak of COVID-19 reminds us that we cannot ignore the prevention and supervision of infectious diseases at any time. Once it happens, the consequences are too serious.

### The number of beds per 10,000 population

We use the number of beds per 10,000 population as the health service supply, for the hospitals in China are always good at expanding, which performed in beds expansion. However, few studies have compared the number of beds per 10,000 population with PTHE per capita. BEDS refers to health supply. We have assumed a positive impact for more beds usually means more inpatients, and therefore, more hospitalization expenses. And our results showed a strong relationship between them. From the director’s point of view, they would like to enlarge their hospital scale, and therefore occupying the market to attract more patients. Which highlights the need to monitor continuously and evaluate rigorously the impact of ongoing number of beds reform in China.

### Conclusion and policy implications

This analysis utilized a pooled panel data for China’s provinces over the period 2009–2016 to examine the determinants of PTHE per capita. The regression results showed that the number of beds per 10,000 population explains most of the variation in per capita PTHE, followed by mortality and GDP per capita. And a negative correlation was found for OOP/THE with PTHE per capita. POP65, the incidence of infectious disease, and THE/GDP with little or no impact on PTHE per capita.

Based on these findings, the following policy implications may be drawn. First, the significant positive impact of health supply implies that a stronger focusses on supply-side drivers of the growth in spending, which remains an effective strategy to deliver high quality care at an acceptable cost. It is a necessity to pay attention to the future long-term impact of the expansion of hospitals manifested in a number of beds. Second, increases in PTHE is related to better “baseline” health level, more PTHE should be given to ensure basic health services in all provinces, and enable them to spend more on health goods and services. But proper supervision and governance must be upheld for appropriate and efficient use of provincial health funds. Third, the infectious disease implies that reforms that constrain growth in infectious disease have achieved some results, as we do not get a positive correlation. But this does not mean that infectious diseases have not caused an increase in health costs. As a result of the significant effect that is being caused by the COVID-19 pandemic, we are very aware that the prevention of infectious disease is still fundamental to the control of the rapid growth of THE in China. Also, we should be aware that CND will become a new challenge for the growth of THE. Fourth, for population aging, more concerns should be given to those in poor health payment status and should develop appropriate medical relief policies to help the elderly gain access to necessary health care services.

Finally, as the level of economic differs from provinces, the disaggregated analysis warrants further studies using finer data breakdowns at the province level and classified the provinces according to their economic development. Further, methodological work should be concerned with the inclusion of measures of uncertainty around PTHE estimates. It would be highly informative to be able to analyze a broader spectrum of health care services, in particular, the long-term care sector, to gain an overall understanding of the influencing factors of THE.

Our study has several limitations. First, we have collected data about the whole provinces in China, but statistics work in different provinces varies from time to time. So, some influencing variables, such as health insurance, medical technology, are not available in all provinces or lack of certain years’ data, unfortunately. Second, for robust analysis, we have conducted models to testify the regression results, but not all models are identical to the results completely. But the main conclusion is coincident. After all, it is difficult to draw the same conclusion from different methods. Future efforts may employ more econometric methods to validate the results. Third, our study has not considered the disparity of economic development among different provinces. For future research could classify the provinces according to their economic level. Fourth, our model could not identify the smooth spatiotemporal trend, because the maximum observation of this data is only 8, which is too small to consistently fit the temporal trend, although we adopt a spatiotemporal panel data model to solve the spatial temporal dependence at the province and annual level. On the other hand, there is not a well-defined measure to specify the” distance” between provinces, which makes it difficult to study the smooth spatiotemporal trend. Future research could be inclined to spatiotemporal semiparametric autoregressive models to get a more precise result [46, 47]. And we wish to finish it in our continued study.

## Supplementary information

**Additional file 1:** Appendix: Sensitivity analysis. Table1 Panel estimation for GEE. Table2 Panel estimation for GMM.

## Data Availability

The datasets supporting the conclusions of this article are included within the article/tables. The raw data can be requested from the corresponding author on reasonable request.

## Abbreviations

HE: Healthcare Expenditure
THE: Total Health Expenditure
PGTHE: Provincial Government THE
GEE: Generalized Estimating Equation
CPI: Consumer Price Index
GMM: Generalized Method of Moments
OOP: Out-Of-Pocket Payments
GHE: Government Health Expenditure
SHE: Social Health Expenditure
GDP: Gross Domestic Product
OECD: Organization for Economic Co-operation and Development
POP65: Proportion of the Population Aged 65 and Over
OOP/THE: Out of-pocket payments as a percentage of THE
GHE/GDP: Government health expenditure as a share of GDP
MOR: Mortality
DIS: The incidence of infectious diseases
BEDS: The number of beds per 10,000 population
