# Supplementary material for: Study of influential factors of provincial health expenditure -analysis of panel data after the 2009 healthcare reform in China
Source: BMC Health Serv Res. 2020 Jul 1;20:606. doi: 10.1186/s12913-020-05474-1 (PMC7327486; doi:10.1186/s12913-020-05474-1)
Supplement: Supplementary file 1 — Additional file 1: Appendix: Sensitivity analysis. Table1 Panel estimation for GEE. Table2 Panel estimation for GMM. [file 12913_2020_5474_MOESM1_ESM.docx]

**Appendix: Sensitivity analysis**

**Using variables before their log-transformed**

We assess whether the findings in this study are robust when these variables are not log-transformed. Other setting is the same as the original GEE. The results are presented in Table 1. In general, the signs of all the coefficients are identical to those in primary models. And the significance of coefficients remains significant, except for THE/GDP. This could be due to the logarithm of the percentage value is controversial.

Table1 Panel estimation for GEE

| Explanatory variables | Coef. | S. E. | P value | | [95% CI] |
| --- | --- | --- | --- | --- | --- |
| GDP per capita | 0.03 | 0.00 | 0.00 | [0.03 0.04] | |
| OOP/THE | -4.90 | 1.11 | 0.00 | [-6.02 -3.79] | |
| THE/GDP | 6.32 | 5.31 | 0.01 | [1.01 11.63] | |
| POP65 | 4.58 | 5.31 | 0.04 | [-0.72 9.89] | |
| MOR | -37.07 | 12.00 | 0.00 | [-49.07 -25.06] | |
| DIS | -3.73 | 6.43 | 0.12 | [-10.17 2.70] | |
| BEDS | 2.98 | 0.79 | 0.00 | [2.19 3.77] | |
| Number of observations=248 Number of groups=31 | | | | | |

Dependent variable: PTHE per capita

**Employing a Generalized Method of Moments(GMM).**

We assess whether the findings are robust to various methods. Table 2 showed the results of GMM. For the sign of variables, the rate of infectious disease and the number of beds per 10,000 population are not identical to the original model, but the statistical significance is reversed. As for the significance of other variables, mortality is not associated with PTHE which is inconsistent with what we expected. Compared with GMM, we use a spatiotemporal panel data model which could specify some alternative specifications based on spatial panels. For GMM which could not gather some spatial dependence at the province level, we could not ask for complete consistency with the GEE. The results of most independent variables are consistent with the original model.

Table2 Panel estimation for GMM

| Explanatory variables | Coef. | S. E. | P value | | [95% CI] |
| --- | --- | --- | --- | --- | --- |
| In GDP per capita | 0.29 | 0.36 | 0.00 | [0.12 0.40] | |
| In OOP/THE | -0.21 | 1.10 | 0.00 | [-0.39 -0.14] | |
| In THE/GDP | 0.20 | 1.97 | 0.13 | [-0.08 0.26] | |
| In POP65 | 0.13 | 3.43 | 0.04 | [0.01 0.29] | |
| In MOR | -1.20 | 6.60 | 0.58 | [-0.45 0.29] | |
| In DIS | 0.16 | 8.44 | 0.03 | [0.04 1.87] | |
| In BEDS | -0.75 | 0.08 | 0.20 | [-1.92 0.41] | |
| Number of observations=217 Number of groups=31 | | | | | |
| Arellano-Bond test for AR(1):z = -2.66 Pr > z = 0.08 | | | | | |
| Arellano-Bond test for AR(2):z = -2.18 Pr > z = 0.03  Arellano-Bond test for AR(3):z = 1.19 Pr > z = 0.23 | | | | | |
| F(8, 209)=2568.63 Prob > F = 0.00 | | | | | |
| Sargan test of overid. restrictions: chi2(5) =10.72 Prob > chi2 =0.1 | | | | | |

Dependent variable: ln PTHE per capita
